# DEM study on the effect of fiber length and content on the macro-micro mechanical behavior of fiber-sand mixture

**DOI:** 10.1038/s41598-025-34063-7

**Published:** 2026-01-13

**Authors:** Fengling Tan, Guangjing Yin, Pengpeng Zhao, Guocheng Sun, Zhe Li, Zongtang Zhang

**Affiliations:** 1 Research and Design Institute, Sinohydro Engineering Bureau 8 CO.,LTD., Changsha, 410004 Hunan China; 2 Xiangyuan Zhenxing Information Technology Service Co., Ltd, Changsha, Hunan, 410128 China; 3 School of Civil Engineering, Hunan University of Science and Technology, Xiangtan, 411201 China

**Keywords:** Fiber-sand mixture, Shear strength, State parameter, Discrete element method, Mechanical properties, Engineering, Materials science

## Abstract

This paper investigates the mechanical behavior of fiber-sand mixtures (FSM) by comprehensively examining the effects of fiber volumetric content (FC) and fiber length (FL) from both macroscopic and microscopic perspectives using the discrete element method (DEM). A hybrid digital photogrammetry and deep learning approach integrating YOLOv5 and U-Net is developed to rapidly identify particle contours and construct realistic shape databases for fibers and sand grains. Based on these databases, numerical biaxial compression tests are conducted on FSM samples with different FC and FL. The results show that increasing FC and FL significantly enhances the peak and residual stress ratios, leading to increases in peak and residual friction angles of up to 8.5% and 6.2%, respectively. Higher FC values also promote volumetric shrinkage, while longer fibers delay volume contraction by providing additional bridging and rotational restraint. Microscopic analysis reveals that increasing FC reduces sand-sand coordination numbers and increases sand-fiber contacts, while longer fibers contribute to a higher sliding contact ratio and improve internal stability by restricting particle rearrangement. The findings elucidate the micro-mechanisms governing strength improvement and deformation behavior in FSM and provide guidance for optimizing fiber reinforcement strategies in geotechnical engineering applications.

## Introduction

 Mature techniques for soil stabilization and reinforcement are often used to obtain improved geotechnical materials^1^. Soil reinforcement can be achieved by adding continuous strips or sheets of material to the soil (systematically reinforced soil) or by adding short discrete randomly distributed fibers^2,3^. Systematic reinforcement can increase the strength and stabilize the surface soil in certain directions.

Reinforcing soils with randomly distributed short fibers has been a focus of research in the last decades^4–7^. Fibers are more likely to mix with soil, resulting in a random and uniform distribution. This material provides an isotropic behavior compared to geotextiles or geomembranes, which allows the fibers to limit the development of potentially weak structural planes^8^. Fibers have also been used as random reinforcement and have been used in the construction of slopes^9,10^ and embankments^11,12^ to systematically increase the strength in certain directions. Therefore, the engineering properties of fiber-reinforced soils are gradually being emphasized worldwide.

The force and deformation characteristics of soils randomly reinforced with fibers are evaluated mainly by the changes in their shear strength parameters caused by the incorporation of fibers (fiber content, fiber type and fiber shape, etc.), such as biaxial compression tests and direct shear tests. As the use of fibers has increased in recent years, researchers have conducted a lot of research on the effect of randomly distributed fibers on the force characteristics of fiber-sand mixtures (FSM), and fiber materials such as coconut shell fibers^12^ and wasted rubber tires^13,14^ have been widely used. Laboratory tests can analyze the mechanical and deformation characteristics of FSM from a macroscopic perspective in detail, but cannot study its mesoscopic mechanical properties from the particle level. Therefore, it is necessary to use numerical simulation analysis method combined with laboratory tests to carry out a more in-depth study of FSM.

Large number of researchers have tried to obtain soil parameters using the discrete element method (DEM) to explain the mechanisms of mechanical behavior of reinforced sands. Several scholars have used DEM to study the mechanisms of FSM. Tingle^15^ conducted unconfined compression tests on sand specimens with discrete fiber reinforcement using DEM. Xu^16^ performed a DEM analysis of the simple shear behavior of binary sand and gravel mixtures. Lobo-Guerrero et al. used DEM to simulate direct shear tests on mixtures of idealized granular materials and randomly oriented fibers^17^. Although a lot of scholars have conducted numerous studies to investigate the mechanical behavior of FSM, as shown above, the behavior is not fully understood due to the variability of the results and the limited nature of some investigated parameters. There are few studies on the effect of fiber length and different admixtures on the performance of FSM.

To fill this gap, this paper intends to conduct a comprehensive experimental study using the DEM framework to quantify the mechanical influences of fiber contents(FC) and fiber lengths(FL) on FSM. First, realistic fiber and sand particle shapes are extracted via YOLOv5 + U-Net, instead of idealized spheres. Then, a database-driven DEM generation strategy capturing particle morphology distributions. In addition, based on the obtained sand and fiber particle shape data, numerical biaxial compression tests are carried out on FSM samples with different FL and FC. This paper focuses on the numerical analysis and comparative study of the effects of macroscopic and microscopic behavior of FSM.

## Materials and models

### Acquisition of sand and fiber geometries

#### Photogrammetry-based acquisition of mixture images

**Fig. 1:**
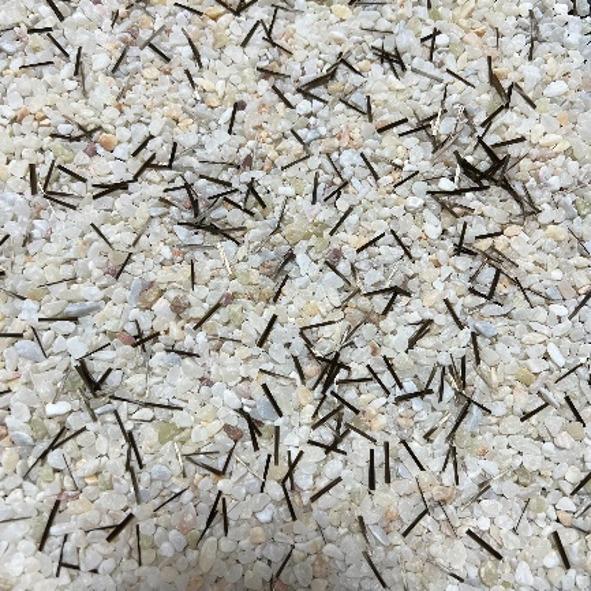
Image of the fiber-sand mixtures.

This section uses digital photogrammetry for the identification and extraction of sand and fiber contours, and builds two shape databases based on statistical analysis. In the laboratory, the sample of FSM is placed under the camera system and its photograph is shown in Fig. 1.

When digital photogrammetry is used to acquire images, the clarity of the images will directly affect whether the recognition results are valid or not. The number of images will affect the overall efficiency of the experiment. Too many images will significantly increase the workload of computer. Insufficient number of images are unable to show related characteristic points on the surface of the particles. Therefore, the key to identification and extraction of sand and fiber contours by digital photogrammetry is to acquire high-quality images with appropriate density. To improve the efficiency of image acquisition, the test steps of Li^18^ are referred to in this experiment. The acquisition of hybrid images based on photogrammetry mainly consists of setting up the camera unit, image acquisition and image post-processing.

The DSLR camera is fixed in the shooting window before taking pictures. The sample is placed in the center of the camera unit for acquisition to reduce the interference of external factors on the photo quality. Appropriate adjustments of the camera parameters are performed to obtain clear images with a considerable depth of field, followed by automatic photography of the mixed samples. A certain degree of overlap between adjacent 2D digital images is required for image acquisition along the specified route, which ensures there are sufficient photographs to cover the entire surface of particles. In the image post-processing part, the commercial software Agisoft Photoscan^19^ is used to output the 2D projection taking point cloud and multi-view stereo (MVS) algorithm methods^20^.

#### Identification of sand and fiber from mixture images

In recent years, deep learning algorithms represented by convolutional neural networks (CNNs) have greatly improved object detection performance. The popular object recognition algorithms are mainly divided into two categories: (1) two-stage algorithms based on detection frames and classifiers, for example, the R-CNN series of algorithms^21^; (2) regression-based first-order algorithms, such as Single Shot MultiBox Detector (SSD)^22^ and YOLO series of algorithms^23^. However, the small size, highly dense and overlapping characteristics of FSM machine images make detection more difficult, and it is difficult to combine both speed and accuracy in the current particle contour detection algorithms.

**Fig. 2:**
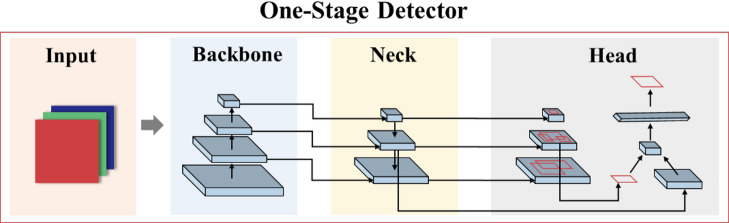
YOLOv5 network structure diagram.

R-CNN series has advantages in target detection with high accuracy, but its detection speed is slow^24–26^. The detection speed of SSD and YOLO algorithms is faster. But in practical scenarios, the detection accuracy of SSD for small objects is not ideal, and it cannot meet the real-time performance of object detection^27^. YOLO algorithms treat image detection as a regression problem, concentrating on global information during training process, which improves the accuracy and detection speed to meet the requirements of target recognition detection. In this study, an improved YOLOv5-based method^28^ is used for contour detection and recognition of sand grains and fibers in FSM. The main components of YOLOv5 can be divided into three parts: input, backbone part, and neck part, as shown in Fig. 2.

The captured images are aggregated by transferring them to a feature extractor (Backbone) and forming image features of different particles. The neck acts as a feature aggregator, blending and combining image features through a series of layers and passing them to the head. The head acts as a feature predictor, consuming features from the neck and taking box and class prediction steps. Finally, the network outputs the target predictions.

To detect sand and fiber, two separate datasets, Y_S and Y_F, are created for training and testing. Each training set contains 500 images (300 for training, 100 for validation, and 100 for testing^29,30^. The U-Net segmentation uses pixel-level feature learning, reducing the risk of sample scarcity. Moreover, the dataset augmentation was employed to increase the effective sample size by 4×. To avoid the training loss caused by dimensional changes, the aspect ratio of all images is set to 1:1.

To judge the accuracy of the detection model, this experiment is evaluated based on the loss function curve (Loss) and the mean average precision (mAP) of the test set results. During the network training process, the loss function can visually reflect whether the model can converge stably with the increase of iterations. In research process, the loss values gradually decrease as the number of iterations gradually increases. all the losses after stabilization are less than 0.03, which indicates that the models are well trained.

**Fig. 3:**
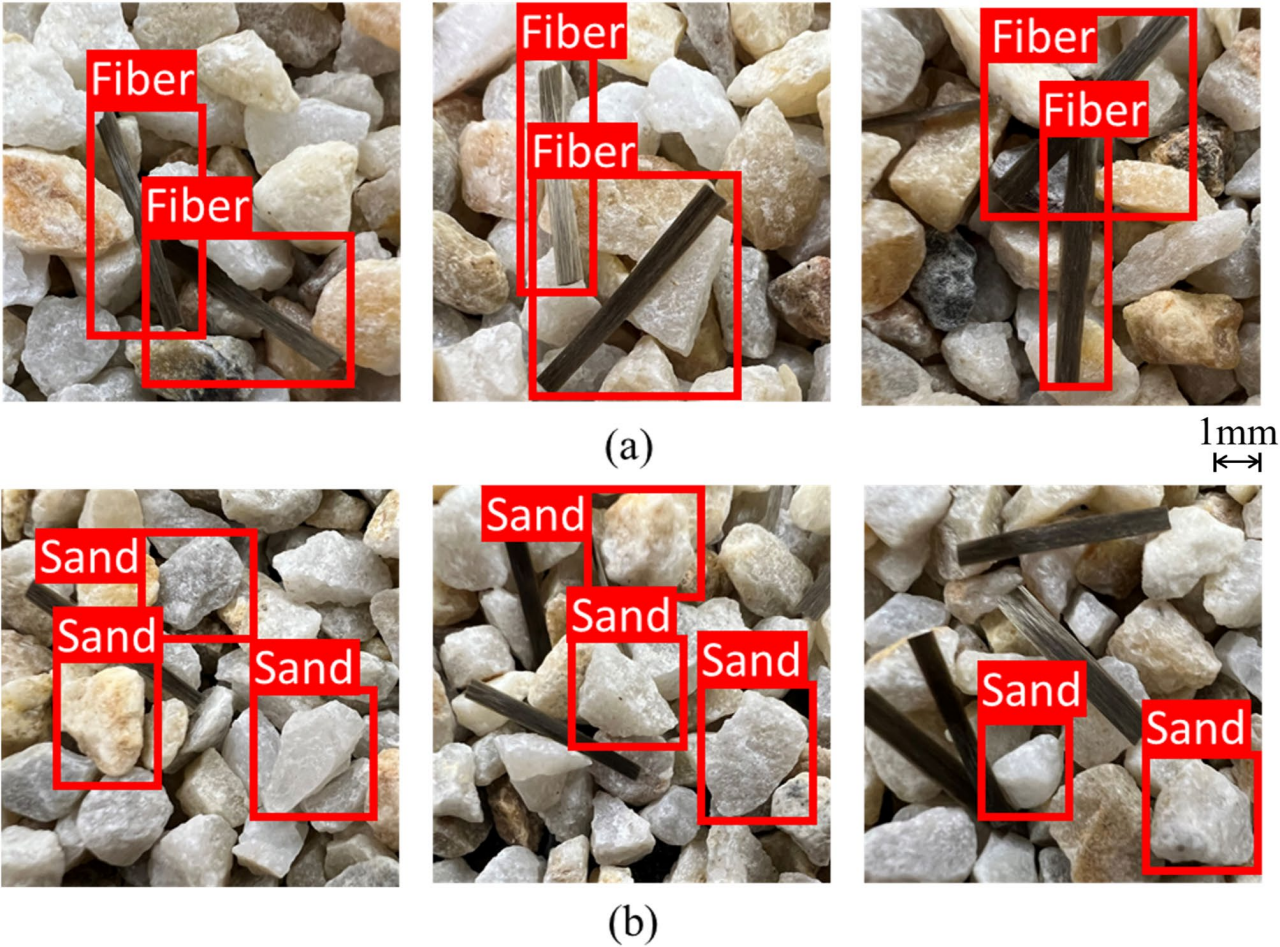
Detection results: (**a**) fiber particle, (**b**) sand particle.

The mAP is used to measure the quality of the detection model. A higher value represents a higher Intersection over Union (IoU). With an IoU threshold of 50%, mAP is greater than 0.88, which further indicates an accurate and reliable model. The detection results are shown in Fig. 3, where both sand and fibers are effectively detected. The detected images are divided into stored into D_S and D_F groups based on the recognition results.

In this experiment, after the YOLOv5-based model discriminated the particles, the U-Net model is used to extract the contours of the particles. The U-Net model classifies the acquired images into pixels, which allows a better feature representation of the segmentation task and achieves a higher segmentation accuracy. Compared with other methods, the U-Net model satisfies the need for high efficiency and high accuracy. Besides, it improves data utilization and supports model training with a small amount of data^31^.

**Fig. 4:**
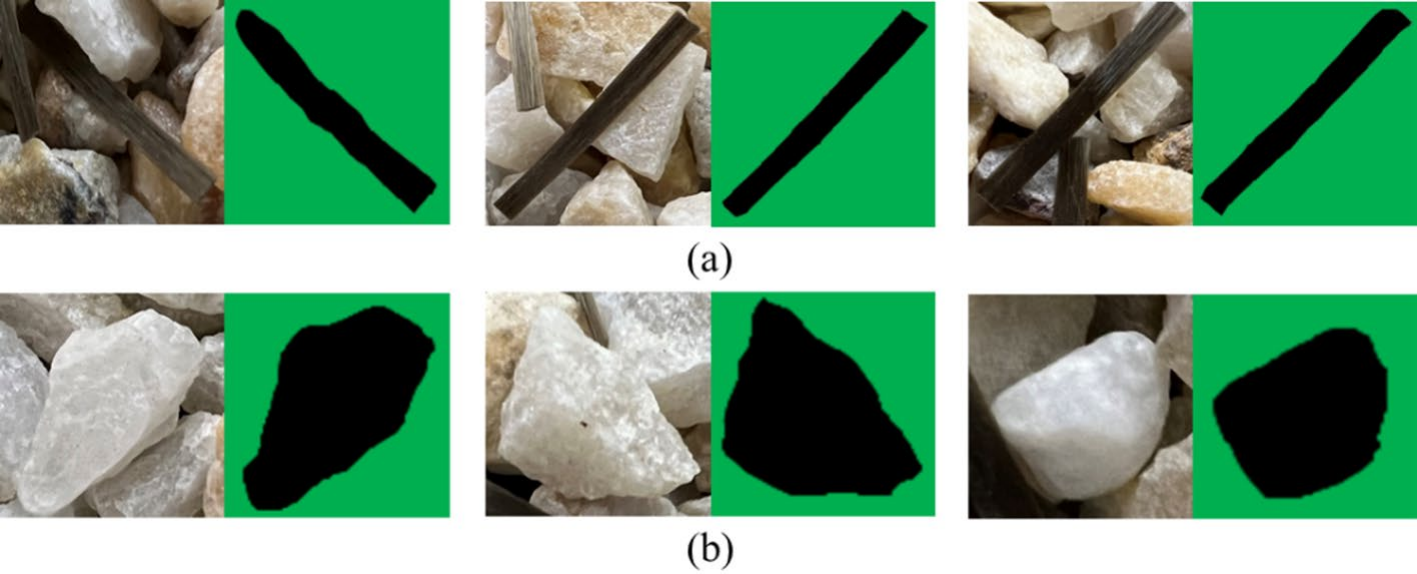
Particle identification results: (**a**) fiber particle, (**b**) sand particle.

Figure 4 shows the recognition results of sand and fiber particles. The recognition results show that the edges of particle contours are clear, which indicates that the image recognition model can effectively recognize and extract the contours of particles, providing convenience for the next numerical simulation experiments. The recognition results of the cropped D_S and D_F groups are stored into C_S and C_F respectively. Then, the shape databases of sand grains and fibers are established. When the particle shape databases are obtained by the above algorithms, each contour document is stored as a file in ‘dxf’ format for the next DEM numerical simulation.

### Modeling of sand and fiber in DEM

In the $\:{\mathrm{P}\mathrm{F}\mathrm{C}}^{2\mathrm{D}}$ model, the basic particle units are assumed to be spheres, and in order to simulate irregular forms of rubber fibers and sand particles, cluster and clump are used to simulate the particle units in this study, respectively.

#### Simulation of sand particle

In the traditional discrete element simulation process, spherical particles are usually used to simulate sand, but spheres can hardly show the interlocking phenomenon of particles. If sand particles are assumed to be spherical, the occlusal friction caused by particle shape will be ignored, which eventually leads to the friction coefficient used in the simulation far exceeding the actual value. Thus, the clumping techniques ‘Clump generate’, ‘Clump attribute’ and ‘Contact cmat default’ are applied to simulate the sand particles considering the interlocking effect, it is necessary to generate clump particles in the biaxial test model, which is an aggregation of pebbles. The relative positions between the constituent pebbles are kept constant. The pebbles outside the clump interact with other particles that they come in contact with, but do not disintegrate themselves.

**Fig. 5:**
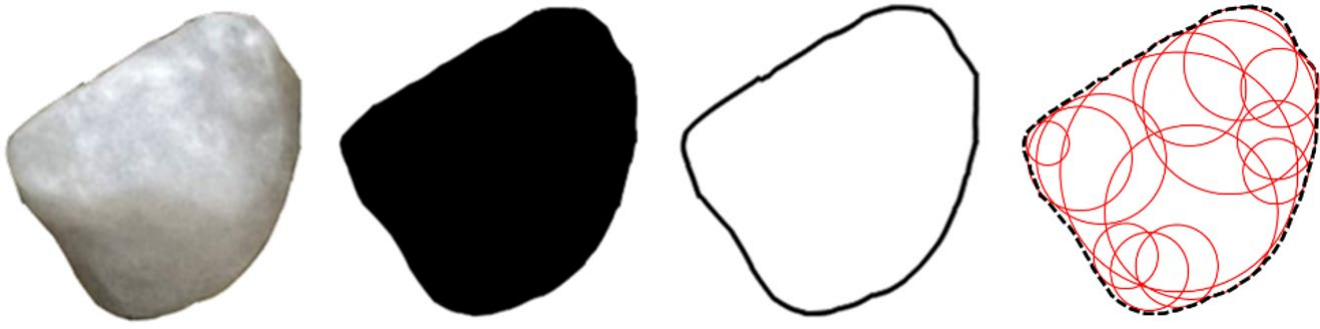
DEM model of sand.

In order to ensure the rationality of the simulation, the intrinsic relationship between the particles is modeled using a linear contact model^32,33^, and the fine view parameters of sand are selected with reference to the literature^34,35^. The normal stiffness is 1 × 10^8^ Pa. The normal-shear stiffness ratio is chosen as 1.25, which satisfies the theoretical model of elastic sphere contact in the Cattaneo-Mindlin model. The particles in the specimen are within the calculated range and meet the calculation criteria. When the $\:{\mathrm{P}\mathrm{F}\mathrm{C}}^{2\mathrm{D}}$ numerical simulation test is carried out, the total number of particles in the mixture specimens under each working condition ranges from 68,312 to 94,105, and the loading system is controlled by servo mechanism to make the numerical simulation process reach the specified surrounding pressure. The clump particles of sand particle unit constructed in this study is shown in Fig. 5.

#### Simulation of fiber

The discs (ball) of the fiber specimen are combined by means of cluster. The fibers are simulated using a bonded particle model, and the fibers are generated as agglomerates (deformed agglomerates) during the wall servo stage to ensure that the fibers do not deform during compaction^36^. And the contact friction coefficient is temporarily set to zero to ensure complete dispersion of the material^37,38^. Subsequently, the balls in the aggregate-based fibers are replaced with discs to generate the fibers. The parallel adhesion model is set between the discs to simulate the viscoelastic mechanical properties of the rubber material^39^.

**Fig. 6:**
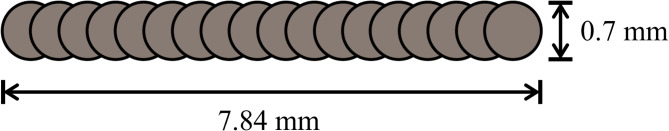
DEM model of rubber fiber.

There are 3 types of contacts in the fiber-sand mixture. Contact between rubber fibers, contact between rubber fibers and sand matrix, and contact within the sand particles. The linear contact model is used to describe the interaction between rubber fibers and sand particles as it is simple and effective in modeling non-cohesive granular materials. In the PFC numerical simulation, the fibers are composed of 2, 18, and 36 particles (the radius of bonded particles is 0.7 mm), respectively. The distance between the centers of circles of two adjacent bonded balls is 0.6 times the diameter. The rubber fiber model in the numerical biaxial experiment is shown in Fig. 6.

The density of the rubber fiber particles is $\:1150kg/{m}^{3}$^40^, the normal stiffness is 1 × 10^8^$\:Pa$ for sand and 1 × 10^7^$\:Pa$ for rubber fiber^41^, and the normal-shear stiffness ratio is chosen to be 1.25 considering the Poisson’s Ratio^42^. Moreover, the friction coefficients are selected from previous studies based on sliding tests. The specific parameters of the rubber fibers and sand particles are shown in Table 1.

**Table 1:** Parameters of fiber-sand mixtures materials in DEM simulation.

Materials	Density $\:\left(\boldsymbol{k}\boldsymbol{g}/{\boldsymbol{m}}^{3}\right)$	Friction coefficient	Normal-to-shear stiffness	Parallel bond normal stiffness$\:\left(\boldsymbol{P}\boldsymbol{a}\right)$	Normal stiffness$\:\left(\boldsymbol{P}\boldsymbol{a}\right)$	Damping coefficient
Fiber	1150	0.6	1.25	6.7 × 10^9^	1 × 10^7^	0.7
Sand	2600	0.3	1.25	-	1 × 10^8^	0.7
Wall	-	-	1.25	-	1 × 10^9^	0.7

## Discrete element modeling of fiber-sand mixtures

### Simulation of DEM-based biaxial compression tests

#### Simulation process

Since the present simulation experiment aims to investigate the effects of FC and FL on the performance of reinforced sand. In order to ensure the reliability of the experiment, the parameters in the model refer to earlier related studies^43–46^. 10 sets of numerical experiments are conducted in this study. A total of four different groups of FC (0% 10% 20% 30%) and four different groups of FL (0 ball, 2 balls, 18 balls, 36 balls) samples are subjected to numerical biaxial compression tests in this experiment^37,47^. The shapes of sand grains and fibers in the simulated experiments were randomly selected from the shape databases C_S and C_F. In addition, to simplify the representation of experiments under different conditions, each group of experiments is identified by an abbreviation. For example, C20L18 indicates an experiment with 20% fiber volumetric content and a fiber length consisting of 18 overlapping balls.

The parameter settings are kept consistent for all simulation groups, and after calibration, the specimen abbreviations and sample information for all experimental groups are listed in Table 2.

**Table 2:** Specimen details in DEM simulation.

Specimen abbreviations	Fiber volumetric content (%)	Fiber length (Balls)
C0L0	0	0
C10L02	10	2 (1.12 mm)
C10L18	10	18 (7.84 mm)
C10L36	10	36 (15.40 mm)
C20L02	20	2 (1.12 mm)
C20L18	20	18 (7.84 mm)
C20L36	20	36 (15.40 mm)
C30L02	30	2 (1.12 mm)
C30L18	30	18 (7.84 mm)
C30L36	30	36 (15.40 mm)

**Fig. 7:**
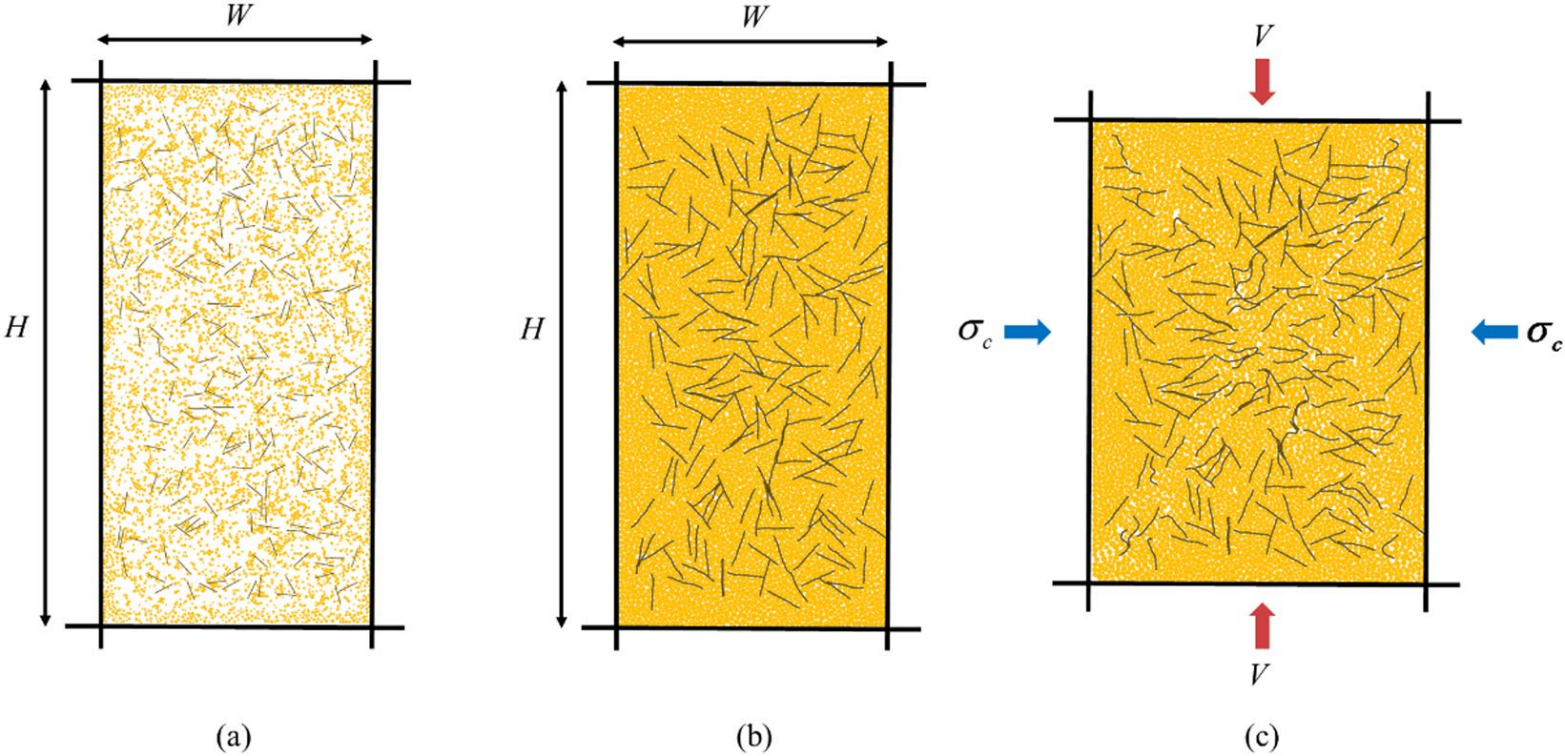
Simulation process of sample generation: (**a**) sample generation, (**b**) wall-servo packing, (**c**) shearing.

In $\:{\mathrm{P}\mathrm{F}\mathrm{C}}^{2\mathrm{D}}$, biaxial compression test. The simulation process is shown in Fig. 7. The process of numerical biaxial compression test consists of three stages^37^. The details of the simulation process are as follows:.

**Fig. 8:**
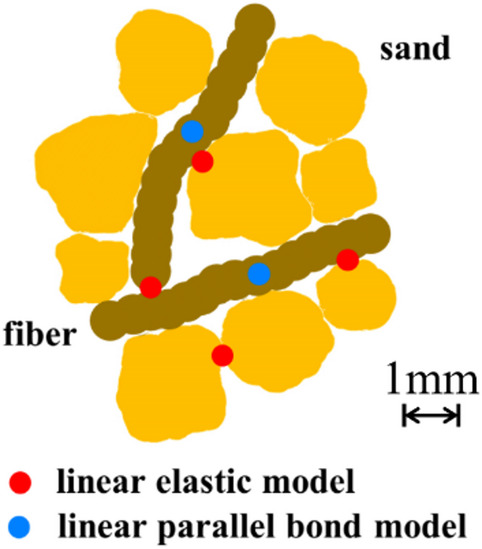
Contact model in fiber-sand mixtures.

In the sample generation, four rigid loading walls are generated, two loading walls and two sidewalls. The loading walls enclose a rectangular frame with an aspect ratio of 2:1. Particles and fibers will be generated randomly in the rectangular frame generation, and the size of the sand particles is set between 1 mm and 2 mm in order to ensure experimental accuracy and efficiency. The particles are then compressed and serviced isotropically^41^ by the loading walls, and to keep the box shape essentially constant, the vertical loading walls are moved with respect to each other at 0.05 and the sidewalls are moved at half the speed of the vertical loading walls to obtain quasi-static behavior. At this stage, the fibers form agglomerates (variable formation agglomerates) to ensure that the fibers are not deformed during compaction. The contact friction coefficient is temporarily set to zero to ensure complete dispersion of the material^36^. In addition, the linear elastic contact model shows the realistic behavior of granular materials with poloidal (e.g., agglomerates and clusters) due to its stable and efficient operation^41^. Therefore, the linear elastic model is adopted for the interaction between sand-sand, sand-fiber and fiber-fiber boundaries, as shown in Fig. 8. Subsequently, the pebbles in the agglomerate-based fibers were replaced with ball-generating fibers. Finally, the sidewalls were removed, an applied peritectic pressure was applied to the particles, and the actual friction coefficient was applied to the particle system and sheared until the axial strain of the sample reached 15%^37^.

#### Variable definitions

The macroscopic stress tensor for the microscopic quantities of contact forces and contact vectors is as follows^48^:

1 $$\:{\sigma\:}_{ij}=\frac{1}{V}\sum\:_{c=1}^{{N}_{c}}{f}_{i}^{c}{d}_{j}^{c}$$

where $\:V$ is the sample volume, $\:c$ denotes a specific contact, $\:{N}_{c}$ indicates the total number of contacts in the sample, $\:{f}^{c}$ represents the corresponding contact force, and $\:{d}^{c}$ is the corresponding branch vector joining the centers of the two particles in contact.

The effective mean ($\:{p}^{{\prime\:}}$) and deviatoric ($\:q$) stresses are defined as^49^:

2 $$\:{p}^{{\prime\:}}=\frac{{\sigma\:}_{1}+{\sigma\:}_{2}}{2}$$

3 $$\:q=\frac{{\sigma\:}_{1}-{\sigma\:}_{2}}{2}$$

where $\:{\sigma\:}_{1}$ and $\:{\sigma\:}_{2}$ represent the axial stress and lateral stress, respectively.

The internal angle of friction, $\:\phi\:$, which represents the shear strength of the granular material, can be defined from the stress ratio in drained biaxial loadings based on the Mohr-Coulomb criterion^50^:

4 $$\:sin\phi\:=\frac{{\sigma\:}_{1}-{\sigma\:}_{2}}{{\sigma\:}_{1}+{\sigma\:}_{2}}=\frac{q}{{p}^{{\prime\:}}}$$

The axial strain, $\:{\epsilon\:}_{1}$, and volumetric strain, $\:{\epsilon\:}_{v}$, are estimated from the boundary movements:

5 $$\:{\epsilon\:}_{1}=\frac{{h}_{0}-h}{{h}_{0}}$$

6 $$\:{\epsilon\:}_{v}=\frac{{v}_{0}-v}{{v}_{0}}$$

where $\:{h}_{0}$ and $\:h$ are the initial and current heights of the sample. $\:{v}_{0}$ represents the initial volume of the sample, and $\:v$ denotes the current volume of the sample.

### Analysis of macroscopic responses

#### Stress ratio and friction angle

**Fig. 9:**
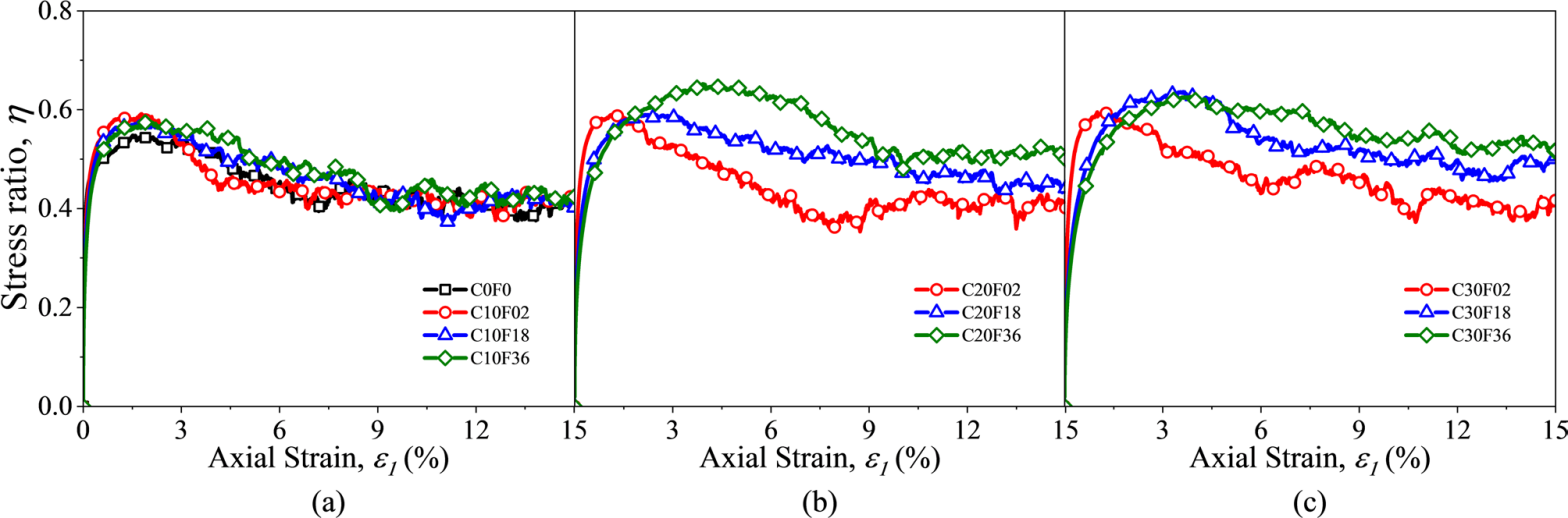
Stress–strain relationship for samples with fiber.

Figure 9 shows the stress ratios during shear for ten groups of FSM with different FL and FC. When the experiment is conducted, the stress ratios first increased rapidly and reached a peak around 3% of the axial strain, then decreased slowly and finally remained near a steady state. The increase in FC resulted in a significant increase in both the peak and residual stress ratios, but the axial strain where the peak point is located appear significantly later. From the picture, it is shown that the addition of fibers makes the initial stage more difficult to compact, indicating that the stiffness of the mixture is altered. From the effect of stiffness it can be seen that the mixture is more loose in the initial stage after fiber addition. At low fiber content, the effect of increasing FL on the stress ratio is not significant. As FC increased, the increase in FL improved the stress-strain relationship of the FSM and improved its strength. When FC was kept constant, the longer the fiber was, the greater the increase in stress ratio was. However, when the fiber length reached a certain level, the effect of continuing to increase the fiber content was no longer apparent. This strengthening effect is consistent with the phenomenon found in previous studies of FSM^15,51^.

**Fig. 10:**
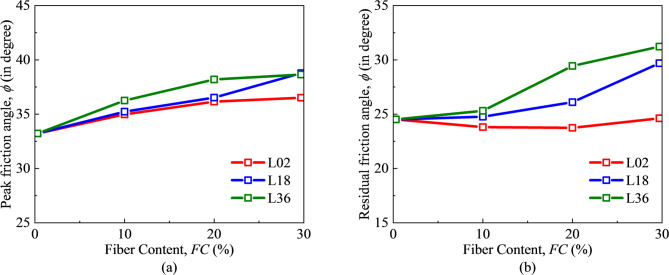
Internal friction angle: (**a**) peak friction angle, (**b**) residual friction angle.

Figure 10 shows the effect of FC and FL on the peak and residual friction angles. As the FC increases, the peak friction angle also rises gradually, and the longer the FL, the greater the difference in the peak friction angle. This is in accordance with the relationship of stress ratio mentioned above. The residual friction angle also basically has a positive relationship with FC. However, at the fiber length of L02, the variation of the residual friction angle with FC is not obvious. The above results show that for FSM, the friction angle increases with the increase of FC and FL. And the increase of FL will help to enhance the effect.

#### Volumetric strain

**Fig. 11:**
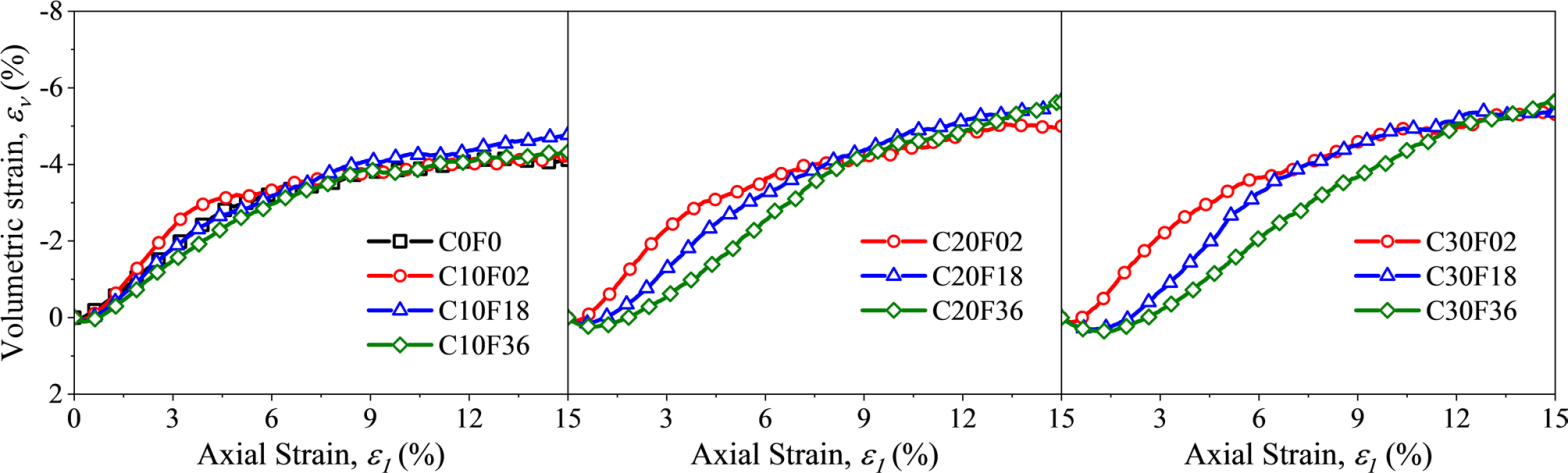
Evolution of the volumetric strain with the axial strain.

Figure 11 shows the variation of volume strain with axial strain for different FL and FC conditions. Ten sets of experimental samples first showed a slight shrinkage with increasing strain, and then the volume strain all gradually decreased until finally reaching the minimum value. Moreover, longer fibers bridge between multiple sand particles, delaying rearrangement and slowing volumetric reduction; Thus, the longer the Fiber, the slower the decreasing trend of the volume strain, but the effect on the final volume strain magnitude was not obvious. The volume strain of FSM decreased when FC increased from 10% to 30%, which could be a shift of the support structure from sand support to sand and fiber co-support. That is, higher fiber content introduces more constraints, enhancing particle confinement and suppressing dilation. This phenomenon suggests that the increase in FC as well as FL led to an increase in volume shrinkage of FSM.

### Analysis of microscopic quantities

#### Coordination numbers

The coordination number $\:Z$ is an important indicator used to study the internal structure of a mixture, and it can be defined according to the contact type. To analyze the contact and interaction forces in FSM, two different types of contacts were considered in this experiment, i.e., sand-sand particles(SS contact), sand-fiber particles(SF contact), and fiber-fiber contact was not considered due to the small number of fibers in this experiment. The coordination number $\:{Z}_{SS}$ of sand-sand particle and $\:{Z}_{SF}$ of sand-fiber particle obtained from the experiment are shown below^52,53^:

7 $$\:{Z}_{SS}=\frac{2{N}_{c}^{SS}}{{N}_{S}}$$

8 $$\:{Z}_{SF}=\frac{{N}_{c}^{SF}}{{N}_{F}}$$

where $\:{N}_{c}^{SS}$ is the total number of sand particles around the sand particles, $\:{N}_{S}$ is the number of sand particles, $\:{N}_{c}^{SF}$ is the number of fibers around the sand particles, and $\:{N}_{F}$ is the number of fibers.

**Fig. 12:**
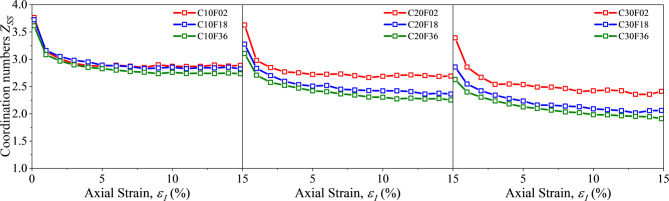
Coordination number $$\:{Z}_{ss}$$.

Figure 12 shows the coordination numbers $\:{N}_{c}^{SS}$ and $\:{Z}_{SF}$ in the FSM mixes. when the axial strain is less than 5%, $\:{N}_{c}^{SS}$ decreases rapidly with increasing strain and reaches a steady state, and then $\:{N}_{c}^{SS}$ decrease slowly during shear. As the FC increases from 10% to 30%, the $\:{N}_{c}^{SS}$ gradually decreases, which indicated that the particles are rearranged during the shearing process and the presence of fibers make the contact between sand particles less tight. The larger the FL, the more pronounced the loosening, which is consistent with the previous study.

**Fig. 13:**
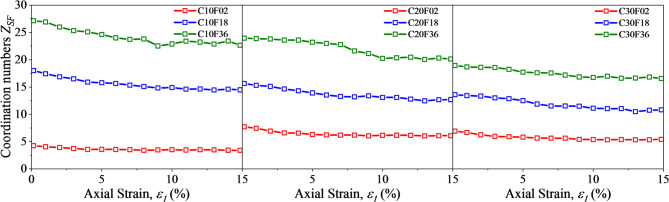
Coordination number $$\:{Z}_{SF}$$.

It is important to note that $\:{Z}_{SF}$ varies with the increase of FC and FS. As shown in Fig. 13, when increasing fiber length, $\:{Z}_{SF}$ increases steadily, which is consistent with the knowledge of the contact between different lengths and particles in space. However, for the shortest fiber F02, when FC increases from 10% to 20%, the change of fiber contents leads to more contact, and the coordination number increases. While it is almost the same when FC rise from 20% to 30% constantly. Meanwhile, for the longer fibers, $\:{Z}_{SF}$ tends to decrease due to the increase of the content. The reason for this phenomenon is that a sand particle may be in contact with multiple fibers, which reduces $\:{Z}_{SF}$, and the longer the fiber length, the more obvious the reduction, which is consistent with the spatial physical phenomenon.

#### Sliding contact percentage

Sliding contact is closely related to Coulomb’s law of friction, and the sliding contact ratio is commonly used to study the interaction between different objects. The FSM mixture in this experiment is a binary mixture, which has been mentioned in Sect. 3.3.1. Therefore, the sliding contact ratio $\:{S}_{SF}$ is calculated as follows^52^.

9 $$\:{S}_{SF}=\frac{{N}_{\mathrm{C}\mathrm{S}}}{{N}_{C}}\times\:100\mathrm{\%}$$

where $\:{N}_{\mathrm{C}\mathrm{S}}$ is the number of sliding contacts in the sample.

**Fig. 14:**
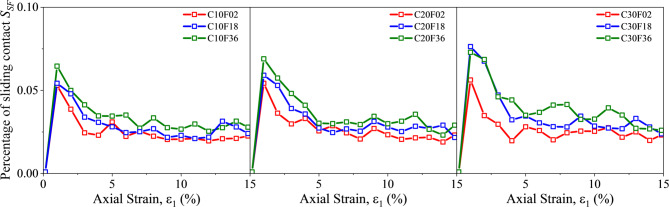
Percentage of sliding contact $\:{S}_{SF}$.

After applying the external load, $\:{S}_{SF}$ rapidly spikes to the peak with increasing strain as shown in Fig. 14. The peak increases with the increase of FC and the increase of FL also contributes to the increase of the peak. Then $\:{S}_{SF}$ decreases and stabilizes, which is consistent with the previous study^54^. The increase in sliding contact percentage means an increase in particle rearrangement^55^, which means that fibers limit particle rotation by increasing the number of contacts, while forcing particles to move with fiber deformation. Contact sliding tends to occur along the interface between surrounding and non-surrounding particles. Therefore, the higher the fiber content and the longer the fiber, the more significant this phenomenon becomes. After the axial strain exceeds 10%, the sliding contact ratio remains unchanged during shear. It is noteworthy that this finding is due to the fact that the addition of fibers limits the movement of the sand, while the high friction between the sand and fibers induces more contact points to slide to accommodate the imposed deformation^56^. This phenomenon is mainly due to the physical phenomenon in space. When the length of the fiber is longer, the effect of resisting rotation and sliding is more obvious, so it is more difficult to slide or rotate.

## Conclusion and discussion

This paper investigates the effect of using discrete randomly distributed rubber fibers in sand particles on the mechanical behavior of fibrous sand mixtures. In this paper, ten sets of numerical biaxial experiments were simulated using a DEM framework, hybrid image acquisition based on photogrammetry, and deep learning methods to identify and extract particle profiles. The experimental results analyze the strength and strength parameters of fiber-sand mixtures from macroscopic and microscopic perspectives, further illustrating the reinforcement mechanism of fibers on fiber-sand mixtures. The main conclusions obtained from the study are:

(1) This paper collects images based on photogrammetry, and uses YOLOv5 and U-Net models to distinguish and contour the particle information in the collected images. The test shows that this system can meet the high-precision requirements of the test and improve the image processing efficiency of multiple images.

(2) The strength of the fiber-sand mixture is improved by the reinforcement of the fibers. The presence of fibers increases the stress ratio and friction angle of the fiber-sand mixture. In addition, the increase in fiber content and fiber length increases the contact surface between the sand particles and fibers, allowing the fibers to support the surrounding sand particles.

(3) Fibers increase the volumetric shrinkage of the fiber-sand mixtures. With the change of coordination number, the volume change of the sample during the shearing process gradually changed from volume expansion to volume contraction. The higher the fiber content, the greater the volume shrinkage of the specimen, and the longer the fiber, the slower the shrinkage. However, the effect on the final volumetric strain is not obvious.

(4) In terms of microscopic properties, the addition of fibers restricts the movement of sand particles, and the longer the fibers are, the stronger the anti-slip effect is, which effectively improves the sliding ratio and enhances the internal stability of the sample.

## Data Availability

The datasets used and analyzed during the current study are available from the corresponding author upon reasonable request.
